# Ascorbic acid attenuates the pressor response to voluntary apnea in postmenopausal women

**DOI:** 10.14814/phy2.12384

**Published:** 2015-04-23

**Authors:** Brittney J Randolph, Hardikkumar M Patel, Matthew D Muller

**Affiliations:** Pennsylvania State University College of Medicine, Penn State Hershey Heart and Vascular InstituteHershey, Pennsylvania

**Keywords:** Aging, blood pressure, heart rate, sympathetic nervous system, vascular resistance, vitamin C

## Abstract

We recently demonstrated that postmenopausal women have an augmented blood pressure response to voluntary apnea compared to premenopausal women. Both obstructive sleep apnea (OSA) and healthy aging are associated with increased oxidative stress, which may impair cardiovascular function. Restoring physiological responses could have clinical relevance since transient surges in blood pressure are thought to be an important stimulus for end-organ damage in aging and disease. We tested the hypothesis that acute antioxidant infusion improves physiological responses to voluntary apnea in healthy postmenopausal women (*n* = 8, 64 ± 2 year). We measured beat-by-beat mean arterial pressure (MAP), heart rate (HR), and brachial artery blood flow velocity (BBFV, Doppler ultrasound) following intravenous infusion of normal saline and ascorbic acid (∼3500 mg). Subjects performed maximal voluntary end-expiratory apneas and changes (Δ) from baseline were compared between infusions. The breath hold duration and oxygen saturation nadir were similar between saline (29 ± 6 sec, 94 ± 1%) and ascorbic acid (29 ± 5 sec, 94 ± 1%). Ascorbic acid attenuated the pressor response to voluntary apnea (ΔMAP: 6 ± 2 mmHg) as compared to saline (ΔMAP: 12 ± 2 mmHg, *P* = 0.034) and also attenuated forearm vasoconstriction (ΔBBFV: 4 ± 9 vs. −12 ± 7%, *P* = 0.049) but did not affect ΔHR. We conclude that ascorbic acid lowers the blood pressure response to voluntary apnea in postmenopausal women by inhibiting vasoconstriction in the limb vasculature. Whether ascorbic acid has similar effects in OSA patients remains to be prospectively tested.

## Introduction

At the physiological level, both obstructive sleep apnea (OSA) (Leuenberger et al. 1995; Somers et al. 1995; Zou et al. 2004) and voluntary apnea during wakefulness (Heistad et al. 1968; Hardy et al. 1994; Leuenberger et al. 2001; Khayat et al. 2004; Steinback et al. 2010) lead to a reduction in arterial oxygen saturation (SaO_2_), a rise in end-tidal CO_2_ (EtCO_2_), and a surge in muscle sympathetic nerve activity (MSNA) (i.e., chemoreflex). This results in vasoconstriction in the limb and renal vascular beds, and the subsequent rise in arterial blood pressure (BP). A recent study from our laboratory indicated that postmenopausal women have augmented BP responses to voluntary apnea compared to young, premenopausal women (Patel et al. 2014). Attenuating the acute pressor response to apnea-induced hypoxemia may be beneficial as transient surges in BP are thought to be an important stimulus for end-organ damage in hypertensive OSA patients (Narkiewicz et al. 2005b). Further, reducing the high sympathetic drive that is present in OSA patients even during wakefulness could have clinical implications (Carlson et al. 1993).

Epidemiological studies have consistently demonstrated that OSA is more common in postmenopausal women than premenopausal women with estimates ranging from 4 to 47% (Dancey et al. 2001; Lin et al. 2008). Moreover, postmenopausal women have an almost fourfold higher risk of OSA, even after adjusting for several confounders such as age and BMI (Bixler et al. 2001; Young et al. 2003), which suggests menopause is an independent risk factor for the development of OSA. Accumulating evidence has shown that both OSA and menopause are associated with an increase in oxidative stress and impaired vascular function (Grebe et al. 2006; Moreau et al. 2007). Indeed, Moreau et al. (2007) found that postmenopausal (i.e., estrogen deficient) women have lower femoral vascular conductance at rest and this effect could be attenuated by acute intravenous infusion of ascorbic acid (Vitamin C), a potent antioxidant (Frei et al. 1989; Levine et al. 1996b). Our group has also shown that ascorbic acid infusion can improve physiological function in some (Gao et al. 2012; Muller et al. 2012) but not all (Muller et al. 2013a; Pollock et al. 2014) studies.

There are currently no data regarding how ascorbic acid affects the physiological responses to apnea-induced hypoxemia in postmenopausal women. Therefore, we tested the hypothesis that ascorbic acid attenuates the pressor response to maximal end-expiratory voluntary apnea (MEEVA) in estrogen-deficient postmenopausal women (i.e., ascorbic acid would modify the efferent arm of the chemoreflex). Because we expected ascorbic acid to lower the pressor responses to apnea via inhibition of sympathetic vasoconstriction, we measured beat-by-beat brachial blood flow velocity (BBFV, Doppler ultrasound) as an index of vasoconstriction in skeletal muscle.

## Methods

### Design and subjects

This study used a repeated measures design, cross-over design in which each subject received both saline and ascorbic acid infusions on the same day. The Institutional Review Board of the Penn State Milton S. Hershey Medical Center approved all study protocols and written informed consent was obtained from each participant.

Eight healthy postmenopausal women (64 ± 2 years, 59–73 years) were studied; each had been without menses for at least 1 year. This sample size was determined once the first four subjects had completed testing. Specifically, we determined that if the mean difference in ΔMAP in response to apnea between saline and ascorbic acid was 5 mmHg with a standard deviation of 4 mmHg we would need to enroll seven subjects to be able to reject the null hypothesis with a probability of 0.80 and a type 1 error of 0.05. All of the participants were normotensive, nonobese, nonasthmatic, and nonsmokers (Table1). Their height (167 ± 2 cm), weight (64.3 ± 1.9 kg), and body mass index (23.1 ± 0.8 kg/m^2^) were within normal limits. Total cholesterol was 180 ± 6 mg/dL (range 149–203 mg/dL), high-density lipoprotein cholesterol was 67 ± 4 mg/dL (range 53–83 mg/dL), and triglycerides were 65 ± 6 mg/dL (range 43-94 mg/dL). Complete blood count values were all within normal limits and all subjects were in good health as determined by history and physical examination. None of the participants were taking hormone replacement therapy or any other medications. Some of the subjects took daily multivitamins but none were consumed on the morning of the study. All of the participants regularly engaged in physical activity (e.g., walking, swimming, volleyball, gardening) but none were competitive athletes.

**Table 1:** Hemodynamic and blood markers in response to saline and ascorbic acid.

	Blood draw 1	Blood draw 2	Blood draw 3	Blood draw 4
	Before NSS	After NSS	After AA	End of AA
Systolic BP (mmHg)	116 ± 3	119 ± 4	122 ± 4	–
Diastolic BP (mmHg)	72 ± 2	74 ± 3	75 ± 2	–
MAP (mmHg)	87 ± 2	89 ± 3	92 ± 3	–
HR (beats/min)	59 ± 2	58 ± 2	59 ± 2	–
FBF (mL/min)	34 ± 5	37 ± 4	37 ± 4	–
MV (L/min)	–	6.72 ± 0.36	7.13 ± 0.44	–
EtCO_2_ (mmHg)	–	38 ± 2	38 ± 2	–
hs CRP (mg/dL)	1.2 ± 0.6	1.1 ± 0.5	1.1 ± 0.5	1.2 ± 0.5
Fibrinogen (mg/dL)	315 ± 16	303 ± 14	307 ± 14	323 ± 14
Apo-B (mg/dL)	71 ± 4	72 ± 5	71 ± 4	72 ± 4

NSS, normal saline solution; AA, ascorbic acid; MAP, mean arterial pressure; HR, heart rate; FBF, forearm blood flow; MV, minute ventilation; EtCO_2_, end-tidal carbon dioxide; hs CRP, high sensitivity C-Reactive Protein; Apo-B, apolipoprotein B. Means ± SE. There were no significant differences between any of the listed time points.

### Protocol

**Figure 1:**
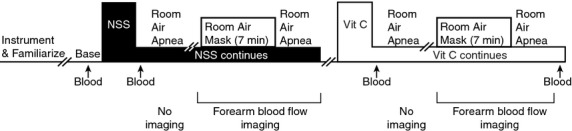
Timeline. NSS = normal saline solution, Vit C = ascorbic acid, base = baseline, the arrows denote that a venous blood sample was obtained.

The studies were performed in a dimly lit temperature controlled (22–25°C) human research laboratory with subjects in the supine position. All measurements were performed in the morning hours after a 24-h abstinence from caffeine, alcohol, and exercise and the participants were in a fasting state (i.e., 8–12 h after their last meal). The subjects wore a high-density tube lined suit (Med-Eng Systems, Ottawa, ON, Canada) that covered the entire body except for the feet, hands, head, and both forearms. Neutral water (34–35°C) was perfused through the suit to maintain mean skin temperature at a constant level and ensure the obtained cardiovascular responses were reflex mediated (i.e., not confounded by ambient temperature). Two intravenous catheters were placed by a registered nurse for the purposes of infusions (left arm) and blood sampling (right arm). Subjects were outfitted with several physiological monitoring devices (see below), practiced the breath hold procedures to ensure they performed expiratory breath holds as previously described (Patel et al. 2013, 2014) and then rested quietly for 15 min prior to baseline hemodynamic measurements and blood sample 1. Figure1 displays the procedures. Note that ascorbic acid was always infused second since it remains in the body for several hours after a high-dose infusion.

After baseline measurements were obtained, 100 mL of normal saline was infused over 20 min and then the infusion rate was lowered (20–30 mL was infused over 60 min). Blood sample 2 was then obtained. The subject performed one MVEEA in room air and only HR, BP, and SaO_2_ were measured during this trial. After a 10 min rest period, the subject was outfitted with an oronasal mask for the measurement of minute ventilation (MV) and end-tidal CO_2_ (EtCO_2_). Seven minutes of room air breathing occurred as HR, BP, SaO_2_, BBFV, TV, and EtCO_2_ were obtained. At the end of the seven minutes, subjects performed a MVEEA. Following a 10 min rest period, ascorbic acid was infused as a loading dose (45 mg/kg in 100 mL of saline) over 20 min. Prior studies in our laboratory have shown this dosing paradigm to alter reflex cardiovascular responses to stress (Muller et al. 2012). A third blood sample was obtained and the ascorbic acid was infused at a lower rate for the remainder of the study (15 mg/kg in 33 mL of saline). Identical MVEEA procedures were repeated during the ascorbic acid infusion. At the very end of the study, a fourth blood sample was obtained.

### Physiological measurements

Beat-by-beat BP was measured by photoplethysmography (Finometer, FMS) and HR was measured by 3-lead EKG (Cardiocap/5; GE Healthcare, Waukesha, WI). MV and EtCO_2_ were determined on a breath-by-breath basis with a respiratory gas monitor (Ohmeda RGM 5250; Ohmeda, Bloomfield, CT). SaO_2_ was measured by ear oximetry (∼3 sec delay). Inspiration and expiration were continuously monitored with a pneumobelt which also allowed for offline analysis of apnea onset and offset. Resting brachial artery BP was obtained in triplicate by an oscillometric device (Philips SureSigns Vs3; Philips, Andover, MA).

Beat-by-beat blood flow was measured in the brachial artery by Doppler ultrasound (GE Vivid 7; GE Healthcare). A 5 MHz linear probe was placed over the left brachial artery (medial to the left biceps brachii) with an angle of insonation <60°. Mean BBFV was measured in pulsed Doppler mode and velocity waveforms were synchronized to a data acquisition system (PowerLab, ADInstruments, Colorado Springs, CO) by a Doppler audio transformer (Herr et al. 2010). Our ultrasound device is not capable of recording simultaneous velocity and diameter measurements, therefore we remained in Doppler mode during the duration of the apnea. We recently demonstrated that brachial diameter does not change in response to MVEEA (Patel et al. 2013). Moreover, test–retest reliability is moderate to strong for ΔMAP, ΔHR, ΔBBFV, breath hold duration, and O_2_ saturation nadir in response to MVEEA (Cronbach's alpha ranged from 0.81 to 0.94) (Patel et al. 2014). Arterial diameter measurements were made at rest and forearm blood flow (FBF) was calculated as cross sectional area × mean BBFV × 60 to express in units of mL/min.

Venous blood samples were drawn at baseline and sent to Penn State Hershey Medical Center clinical laboratory where standard procedures were used to measure complete blood count, high sensitivity C-reactive protein (hs CRP), fibrinogen, apolipoprotein B (Apo-B), and a standard lipid panel. Following the infusion of saline and ascorbic acid, high sensitivity C-reactive protein (hs CRP), fibrinogen, and Apo-B were again quantified (Table1). Apo-B was chosen instead of a standard lipid panel for these repeat measurements since ascorbic acid dramatically influenced our pilot results (*n* = 4), likely due to interference in the measurement assay.

### Data collection and statistical analysis

All data were collected at 200 Hz using PowerLab (ADInstruments, New Castle, Australia) and were analyzed offline. The FBF, MV, and EtCO_2_ were only measured at baseline (i.e., prior to apnea). We quantified the duration of the MVEEA, lowest O_2_ saturation, HR, MAP, and BBFV. Forearm vascular resistance index (FVRI) was calculated as MAP/BBFV. We analyzed four different time points, detailed below:

Base: The last 20 sec prior to beginning the apnea.
Apnea 1 (A1): The first 3 cardiac cycles of the apnea after the subject had completely emptied their lungs (absence of lung inflation but no reduction in SaO_2_ relative to Base).
Apnea 2 (A2): The last 3 cardiac cycles before the subject inhaled (called the “asphyxic break point”) at the end of the apnea (absence of lung inflation and a reduction in SaO_2_ relative to Base).
Recovery 1 (R1): The first 3 cardiac cycles after the subject resumed breathing.

To evaluate the effects of each infusion, paired samples *t*-tests were used. Data are presented as Mean ± SEM and *P* values < 0.05 were considered statistically significant.

## Results

The postmenopausal women received a total of 3832 ± 120 mg of intravenous ascorbic acid during the study. The breath hold duration was similar following saline and ascorbic acid (saline: 29 ± 6 vs. ascorbic acid: 29 ± 5 sec, *P* = 0.679) and the O_2_ saturation nadir was also comparable (saline: 94 ± 1 vs. ascorbic acid: 94 ± 1%, *P* = 0.879). As shown in Table1, ascorbic acid did not influence any of the blood markers that were measured. The FBF, MV, and EtCO_2_ at rest (i.e., the seven minutes prior to onset of MVEEA) were comparable between saline and ascorbic acid (Table1).

**Figure 2:**
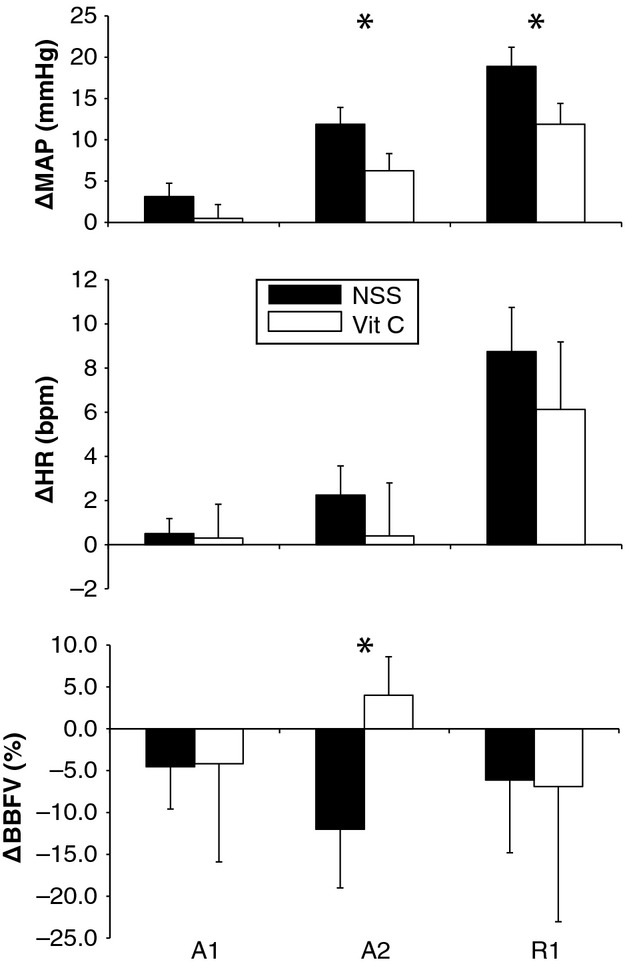
Changes in ΔMAP, ΔHR, and ΔBBFV in response to voluntary apnea in postmenopausal women with NSS (solid bars) and following ascorbic acid (open bars) at the indicated time points. A1 = The first three cardiac cycles of the apnea, A2 = the last three cardiac cycles before the subject inhaled (called the “asphyxic break point”), R1 = the first three cardiac cycles after the subject resumed breathing. Means ± SE, *P <* *0.05 between treatments.

As shown in Fig.2, ascorbic acid blunted the rise in MAP at both A2 (*P* = 0.034) and R1 (*P* = 0.017) compared to saline but had no effect on HR (Fig.2, middle). Ascorbic acid significantly attenuated the reduction in BBFV at A2 (*P* = 0.049, Fig.2 bottom). In a similar way, the percent increase in FVRI at A2 was significantly less following ascorbic acid (2 ± 8%) compared to saline (28 ± 6%, *P* = 0.022). Taken together, the effect of ascorbic acid on the pressor response is partly due to attenuated sympathetic vasoconstriction in skeletal muscle.

When analyzing the trials in which no imaging was performed, ΔMAP at A2 was significantly lower under ascorbic acid (6 ± 1 mmHg) as compared to saline (10 ± 1 mmHg, *P* = 0.007). The ΔHR at A2 was also less under ascorbic acid (3 ± 1 vs. 1 ± 1 beats/min, *P* = 0.036). Both ΔMAP and ΔHR had moderate effect sizes (0.59 and 0.40, respectively).

## Discussion

The purpose of this study was to determine the effects of acute antioxidant infusion (ascorbic acid) on physiological responses to voluntary apnea in healthy postmenopausal women. Consistent with our hypothesis, acute infusion of ascorbic acid blunted the pressor response to voluntary apnea in postmenopausal women in part by attenuating the reduction in skeletal muscle blood flow. These findings may be clinically significant as repetitive surges in BP lead to end-organ damage in OSA patients, and are an associated risk factor for coronary artery disease (Narkiewicz et al. 2005a).

We (Patel et al. 2014) and others (Owens et al. 1993) have previously demonstrated that postmenopausal women have augmented BP responses to sympathetic activation compared to young, premenopausal women. We believe that estrogen deficiency plays a major role in the observed responses. Specifically, estrogen deficiency may influence the transduction of sympathetic nerve activity into vascular resistance (Vianna et al. 2012), thereby contributing to an augmented BP response in postmenopausal women. Age-related impairments in neurovascular control have been previously demonstrated by our group (Heffernan et al. 2013; Pollock et al. 2014) and others (Hogikyan and Supiano 1994; Dinenno et al. 2005). A common mechanism between aging and menopause is oxidative stress, defined as a disturbance in the balance between the production of reactive oxygen species (ROS) and antioxidant defenses. Along with aging, menopause is associated with heightened oxidative stress, as evidenced by reduced total antioxidant status in postmenopausal women (Hildreth et al. 2014). Recent evidence suggests that oxidative stress can directly impair vascular function in healthy postmenopausal women (Moreau et al. 2007; Hildreth et al. 2014) and young men (Fadel et al. 2012). The leading theory is that ROS can directly and/or indirectly decrease nitric oxide (NO) bioavailability (Forstermann 2010) resulting in endothelial dysfunction and a proconstrictor state (Kojda and Harrison 1999). The aforementioned studies examined postmenopausal women under resting conditions, but it is unclear how ROS influence physiological responses during sympathetic stress.

The major finding of the current study is that ascorbic acid attenuates the pressor response to MVEEA, a powerful sympathetic stimulus. By measuring beat-by-beat BP, HR, and BBFV, we determined the BP attenuation is partly mediated by blunted skeletal muscle vasoconstriction with ascorbic acid. Ascorbic acid acutely modifies vascular responses in various conditions associated with heightened oxidative stress such as aging (Taddei et al. 2001), peripheral arterial disease (Muller et al. 2012), and even coronary artery disease (Levine et al. 1996a). When infused at supraphysiological doses, several mechanisms of action have been proposed, including directly scavenging ROS (Frei 1999) and stabilization of BH_4_ (Forstermann 2010), with the end result being increased local NO bioavailability. In this study, the effects of ascorbic acid on the skeletal muscle vasculature are probably due to NO modulation in endothelial cells to indirectly alter forearm vascular resistance index. In the absence of ascorbic acid (i.e., low NO bioavailability), full expression of apnea-induced peripheral vasoconstriction is seen resulting in high FVRI in the postmenopausal women we studied. However, with ascorbic acid, the increased NO bioavailability opposes the peripheral vasoconstriction resulting in blunted FVRI compared to saline. This is similar to the findings of others where ascorbic acid significantly increased muscle blood flow during continuous dynamic exercise (Kirby et al. 2009), and after moderate intensity plantar flexion (Wray et al. 2009). An alternative explanation is that ROS directly influence the carotid body under normal circumstances (i.e., either enhancing or suppressing the chemoreflex) and ascorbic acid blocks this process (Pokorski and Marczak 2003; Del Rio et al. 2010). While our data cannot identify a clear physiological mechanism, the data show that under ascorbic acid the vascular tone balance is tipped toward greater conductance due to less forearm vasoconstriction (Fig.2).

In this study, ascorbic acid did not affect any relevant blood markers such as hsCRP (marker of inflammation), fibrinogen (marker of hemostasis), or Apo-B (marker of lipids) in postmenopausal women. Hildreth and colleagues (Hildreth et al. 2014) recently found that C-reactive protein decreased after ascorbic acid infusion in postmenopausal women. It is worth noting that the postmenopausal women in that study had a BMI of 27.6, compared to our study (23.1), which could explain the discrepancy given obesity is associated with increased oxidative stress (Moreau et al. 2007). While the inverse relationship between chronic serum ascorbic acid levels and fibrinogen levels is clear in epidemiological studies (Langlois et al. 2001; Wannamethee et al. 2006), there is little evidence of changes in fibrinogen or Apo-B markers directly after acute infusion of ascorbic acid.

## Limitations

In the present study, we did not measure serum follicle-stimulating hormone, estrogen, or progesterone, but rather assumed postmenopausal status based on the lack of menses for greater than 1 year. In addition, we cannot determine if the observed responses are due to aging or menopause since there is a tight association between the two variables (Hildreth et al. 2014). However, since each woman served as her own control, we do not believe this affects data interpretation. It is important to note that MVEEA is not a true OSA per se because it does not involve arousal from sleep or upper airway collapse (i.e., both of which engage the sympathetic nervous system) (Chouchou et al. 2013; Muller et al. 2013b; Dempsey et al. 2014). However, we believe there is similar underlying pathophysiology between MVEEA and OSA, allowing us to make useful comparisons between men and women (Imadojemu et al. 2002). Because we did not measure sympathetic nerve activity, it is unclear whether ascorbic acid inhibits nerve activity or alters alpha-adrenergic responsiveness. We also administered ascorbic acid intravenously; thus both the afferent and efferent pathways of the chemoreflex could be affected.

## Conclusions

The present study indicates that in postmenopausal women, acute administration of ascorbic acids blunts the pressor response to voluntary apnea in part due to inhibition of skeletal muscle vasoconstriction. These results extend the findings that oxidative stress contributes in part to augmented pressor responses in older postmenopausal women. Whether ascorbic acid has similar effects in patients with OSA warrants further study.
